# Team sport athletes’ perceptions and use of recovery strategies: a mixed-methods survey study

**DOI:** 10.1186/s13102-017-0071-3

**Published:** 2017-02-24

**Authors:** Fiona Crowther, Rebecca Sealey, Melissa Crowe, Andrew Edwards, Shona Halson

**Affiliations:** 1 College of Healthcare Sciences, James Cook University, Townsville, QLD Australia; 2 Division of Tropical Health and Medicine, James Cook University, Townsville, QLD Australia; 3 Sport & Health Sciences, University of St Mark and St John, Plymouth, Devon UK; 4 Australian Institute of Sport, Canberra, ACT Australia

**Keywords:** Cold water immersion, Contrast water therapy, Stretching/flexibility, Active recovery

## Abstract

**Background:**

A variety of recovery strategies are used by athletes, although there is currently no research that investigates perceptions and usage of recovery by different competition levels of team sport athletes.

**Methods:**

The recovery techniques used by team sport athletes of different competition levels was investigated by survey. Specifically this study investigated if, when, why and how the following recovery strategies were used: active land-based recovery (ALB), active water-based recovery (AWB), stretching (STR), cold water immersion (CWI) and contrast water therapy (CWT).

**Results:**

Three hundred and thirty-one athletes were surveyed. Fifty-seven percent were found to utilise one or more recovery strategies. Stretching was rated the most effective recovery strategy (4.4/5) with ALB considered the least effective by its users (3.6/5). The water immersion strategies were considered effective/ineffective mainly due to psychological reasons; in contrast STR and ALB were considered to be effective/ineffective mainly due to physical reasons.

**Conclusions:**

This study demonstrates that athletes may not be aware of the specific effects that a recovery strategy has upon their physical recovery and thus athlete and coach recovery education is encouraged. This study also provides new information on the prevalence of different recovery strategies and contextual information that may be useful to inform best practice among coaches and athletes.

## Background

There are many post-exercise recovery options currently available for athletes. Some of these include water immersion, stretching (STR), walking and/or jogging, swimming or pool walking, massage, sleeping/napping and fluid/food replacement [1–3]. Although it is generally accepted that many athletes undertake post-exercise recovery, to the authors’ knowledge there is currently no research available to describe which recovery strategies are used by Australian-based athletes across a range of team sports and competition levels. It is also unclear why athletes partake in recovery strategies and if they believe they are effective or ineffective.

Hydration, nutrition and sleep have been reported in the literature as important components of the recovery process [4, 5]. Although used often, further research is required to confirm the effectiveness of STR, active recovery, cold water immersion (CWI) and contrast water therapy (CWT) due to conflicting results reported across randomised controlled trials [6–8] and systematic reviews [1, 9–11].

A survey undertaken by elite South African team sport athletes reported sleep, fluid replacement and socialising with friends as the most popular recovery strategies undertaken [12]. In contrast, STR and CWI were found to be most used by elite South African rugby players (83%), followed by active recovery (74%), with CWI rated most effective [13]. Seventy-nine per cent of surveyed elite New Zealand athletes reported the use of CWT [2]. Interviews with coaches from a state academy of sport in Australia indicated that accessibility and practicality of recovery methods influenced their implementation of different recovery strategies, with the most popular recovery strategies being nutrition, STR, active recovery and CWT [14]. Coaches implemented recovery strategies that they perceived as being effective based on their own past experiences, observations and instinct rather than scientific evidence [14]. Practitioners with French professional soccer teams reported a high prevalence of CWT and CWI use (88% of teams) with active recovery, massage, STR, compression and electrical stimulation used to a lesser extent [4]. Moreno and colleagues [15] found that an individualistic approach to player recovery is required, after Spanish professional basketball players were found to use varying recovery strategies and have different perceptions of them.

These studies provide an insight into the recovery methods used by elite team sport athletes in a limited number of countries, but do not capture sub-elite levels of sports participation and athlete perceptions and reasons for usage of recovery. Furthermore, although Australian coaches’ views on recovery have been reported there appears to be no investigation into the use of recovery strategies by Australian athletes. In response to the widespread use of land and pool-based recovery strategies, yet current uncertainty regarding their effectiveness and reasons for use, this study employed a survey to investigate the recovery techniques used by team sport athletes across various levels of competition (local to international), and who mostly reside in regional Australia. The survey will report if/when, why and how the following five recovery strategies are used: active land-based recovery (ALB), active water-based recovery (AWB), STR, CWI and CWT. This study will also compare the reported reasons for use with available scientific evidence of recovery mechanisms. It is hypothesised that most athletes use recovery, with stretching likely the most popular choice by all levels of athlete, due to its accessibility and because athletes can perform stretching together as a team. It is also hypothesised that CWI and CWT will be considered the most beneficial recovery strategies, due to the high use of these recovery strategies by elite athletes portrayed in the media.

## Methods

To determine the popularity of specific recovery strategies and their reasons for use a survey was deployed that consisted of questions requiring a combination of checkbox, Likert scale and open ended, free text responses. A survey was deployed as it was accessible by a large number of people from different sports. The survey design was based on a combination of previously published surveys on recovery strategies [2, 3, 14]. The survey was available for completion in print, comprised of seven sections, and took approximately 20 min to complete.

**Fig. 1:**
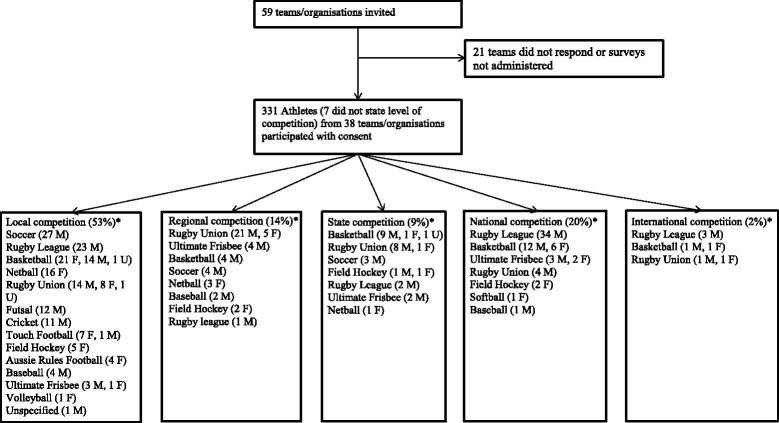
Major sport and level of competition of survey participants. *Participants were allocated according to their highest level of current competition for their dominant sport; F = female, M = male, U = unspecified

Coaches/administrators from a convenience sample of 59 sporting teams/organisations within the northern region of Queensland, Australia were contacted via email or phone to provide consent for their team members to participate in the study. Organisation email addresses and phone numbers were obtained via internet searching or by personal contacts. Competitors from a range of team sports (Fig. 1) across a variety of senior competition levels (excluding social competition) from five cities/towns provided individual consent and completed the survey after a game or training session over a 14 month period between September 2013 and November 2014 (Fig. 1). Players from a metropolitan, capital city basketball college also participated following a snowball invitation by a coach from the survey sampling area. Ethics approval was granted by the Human Ethics Committee James Cook University, Australia and the rights of the participant were protected.

The first section of the survey consisted of demographic information. Participants nominated the major sport that they played and the competition months for that sport, the highest level of competition in which they currently engaged for that sport, the weekly frequency and duration of competition and training, and their age and gender. The second section investigated the recovery strategies employed by the participants. Participants were asked to answer either ‘yes’ or ‘no’ separately to whether they performed a recovery strategy after competition, and/or after pre-season training and/or after in-season training. Participants who did not partake in recovery were invited to explain in free text why they did not, and this concluded their survey participation. Conversely, if participants answered ‘yes’ to any of the three questions about recovery strategy use, they were then invited to select from a predetermined list, [2, 3] the recovery methods that they use after competition, pre-season and/or in-season training; and then in free text to nominate which recovery method they believed to be the most effective. The list of recoveries included; active-land based (ALB), active pool-based (AWB), active stretching cool down (STR), cold/ice bath/shower (CWI), contrast bath/shower (CWT), massage, sleep/nap, food and/or fluid replacement, ice pack/vest application, heat pack application, liniment or gel application, progressive muscle relaxation or imagery, prayer or music, reflexology or acupuncture, supplement use, medication use and other (participants were asked to specify).

A number of recovery strategies such as food, sleep and hydration may be considered to be lifestyle choices that athletes partake post-game/training, but may not be deliberate choices to undertake a specific recovery strategy. In contrast, water immersion recoveries and ACT are recovery choices undertaken with the purpose of recovery, and are choices that can be partaken in as a team and hence were selected to be investigated further. Thus, sections 3-7 of the survey investigated the use of ALB, AWB, STR, CWI and CWT recovery strategies, with one section allocated to each strategy. These strategies were selected based on published research methodologies [2, 3] and the strategies commonly used by Australian sporting teams [1, 14]. The following definitions of recoveries were included in the survey to assist respondents: ALB- includes activities such as or similar to walking, slow jogging, low intensity cycling; AWB- includes activities such as swimming, pool walking, pool jogging; STR - includes static stretching, proprioceptive neuromuscular facilitation stretching, or dynamic stretching (with descriptions included); CWI- includes immersion in cold or ice water; and CWT- includes alternation between immersion in cold/ice water and hot water. In each of these sections participants were asked whether they performed the recovery after competition, after pre-season training and/or after in-season training. If they answered ‘yes’ to any of these questions the participant was directed to answer more questions about that specific recovery strategy. If the participant answered ‘no’ to the three questions they were invited to move to the next section of the survey. The additional questions in each section focused on the perceived effect of each strategy. Participants rated from 1 (not at all) to 5 (very) how effective they considered the recovery strategy to be and were invited to provide a description of why they thought the strategy was effective/ineffective. From a list of twenty potential reasons [2, 3] (Table 1), participants rated how important they thought each reason was for performing the specific recovery strategy from 1 (not important reason) to 5 (very important reason). At the end of each section (sections 3-7) participants were invited to provide specific details about the recovery sessions they undertook (session type, description of recovery, duration and intensity of recovery and how long after the session the recovery was performed).

**Table 1:** Mean participant ratings (1-5) of the importance of different reasons why specific recovery strategies are used; 1 = not important reason; 3 = neither important nor unimportant reason; 5 = very important reason

*Reasons why a recovery is performed (selected from a list of predetermined options)*	Active, land-based recovery (*N* = 82)	Active, water-based recovery (*N* = 100)	Stretching (*N* = 144)	Cold water immersion (*N* = 89)	Contrast water therapy (*N* = 52)
Helps me to wind down and relax	3.5 ^a^	3.8 ^b^	3.7 ^b^	3.3	3.6
Gives me time to socialise with team mates	3.1	3.2	3.3	3.1	3.0
Gives me time to reflect on the training session or match	3.3 ^c^	3.1	3.3 ^c^	3.2	3.0
Makes me feel good	3.4 ^d^	4.0	3.9	3.8	3.8
Is what I have seen the elite athletes do	2.6 ^d^	3.0	3.1	3.4 ^d^	2.9
Is something the coach told me to do	3.3	3.3	3.4	3.4	3.3
Will increase muscle performance	3.2 ^d^	3.6	3.8	4.0 ^d^	3.6
Speeds up removal of waste product from muscles	3.6 ^e^	3.8	3.8	3.9	3.9
Decreases muscle soreness	3.9 ^f^	4.1	4.2	4.1	4.1
Reduces swelling and inflammation	3.4 ^c^	3.6	3.7	4.2 ^d^	3.8
Reduces muscle spasms	3.3 ^a,b,f^	3.8 ^c^	3.8 ^c^	3.7	3.5
Increases blood circulation	3.7	3.8 ^e^	3.6	3.5	3.5
Reduces stress and anxiety	3.2 ^a^	3.7 ^e^	3.4	3.3	3.3
Makes me feel energetic	2.9 ^a^	3.3 ^b^	3.1	3.0	3.2
Can improve healing	3.3 ^d^	3.7 ^b,f^	4.0 ^c^	4.1 ^c^	3.6
Helps me to switch off	3.0 ^a^	3.3 ^b,f^	3.0	3.0	3.3
Helps me to be able to train/compete hard again in the next session/game	3.5 ^d^	3.8	4.1	4.0	4.0
Lowers heart rate	3.2	3.1	3.2	3.1	3.1
Creates a pumping action in the muscles	2.9 ^a^	3.2 ^f^	2.8 ^e^	3.0	3.1

^a^ Significantly different from active, water-based

^b^ Significantly different from cold water immersion

^c^ Significantly different from contrast water therapy

^d^ Significantly different from all other recoveries

^e^ Significantly different from cold water immersion and contrast water therapy

^f^ Significantly different from stretching

### Statistical analyses

A combination of quantitative and qualitative analyses were conducted. The quantitative analysis was conducted on the scale-based ratings data using Statistical Package for Social Sciences (IBM SPSS Incorporation, version 22, Chicago, Ill, USA). The data were found to be approximately normally distributed with the large sample size of 205 in sections 3-7, thus repeated measures ANOVA tests with an alpha set at .05 were conducted to compare ratings across the five recovery strategies. Data were presented as means ± standard deviation (SD) or proportions (%) of responses. Qualitative analysis involved grouping popular responses into specific themes and quoting text directly as specific examples. The identification of themes and allocation of themes was undertaken by two researchers independently. The researchers compared their analysis and together developed the final themes and allocation of responses to themes via consensus.

## Results

Three hundred and thirty-one athletes from 38 teams (71% male, mean age 25 ± 7 years) completed the paper-based surveys. Fourteen team sports and five levels of competition were represented (Fig. 1). Local competition was most represented (53%), followed by national (20%) regional (14%), state (9%) and international (2%). Basketball was the most represented team sport (22%) followed by rugby league and rugby union (20% each), soccer (10%) and netball (6%). Across all sports and levels of competition athletes competed in 0–7 games per week, equating to 0–600 min of competition per week and trained for 0–30 or more hr per week. One competition game, 60 min of competition and 4 h of training per week were the most common responses for the competition and training demographics.

**Fig. 2:**
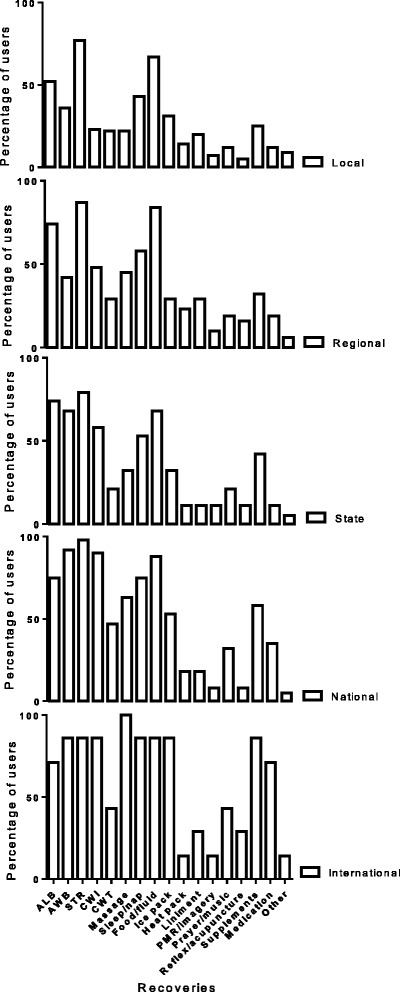
Recovery strategies undertaken by team sport athletes competing in local, regional, state, national and international competition. PMR = progressive muscle relation; reflex = reflexology

Fifty-nine percent of participants self-reported (selected checkbox options) performing a recovery strategy following competition, 55% after pre-season training and 57% used recovery strategies after in-season training. All participants who performed at an international level indicated using massage for recovery (Fig. 2). In contrast the most popular recovery method undertaken by all other levels of athletes (selected checkbox options) was stretching (98% national, 79% state, 87% regional and 77% local) (Fig. 2). Food/fluid (84% regional and 67% local) and ALB (74% regional and 52% local) were the next most popular recovery techniques used by both regional and local athletes (Fig. 2).

Via free text responses sleep (57%) followed by massage (29%) were considered the most effective recovery techniques by the international athletes, while ice bath (55%) and stretching (35%) were considered the most effective by the national athletes. State, regional and local athletes all perceived stretching to be the most effective recovery (32%, 42% and 37% respectively) followed by ice bath (26%, 23% and 14% respectively). Forty-three per cent of athletes reported that they did not participate in post-exercise recovery and of these respondents, self-reported laziness (20%) and time constraints (17%) were the most common reasons provided for not undertaking any post-exercise recovery.

Two-hundred and five athletes completed survey sections 3–7. Across all combined competition levels the athletes that performed STR (4.42 ± 0.61) and CWI (4.3 ± 0.57) rated them to be significantly more effective for recovery than the users of ALB (3.63 ± 0.57, *p* < 0.05), AWB (4.09 ± 0.54, *p* < 0.05) and CWT (4.14 ± 0.41, *p* < 0.05). Active water-based recovery and CWT were also rated significantly more effective than ALB (*p* < 0.05).

The most highly rated reason for use from the predetermined list for ALB, AWB, STR and CWT was ‘decreases muscle soreness’ (Table 1). This is supported by the free-text answers from participants with the most common psychological reason reported for the effectiveness of each recovery being ‘decreases muscle soreness’ (excluding AWB and CWI). For CWI the highest rated reason was ‘reduces swelling and inflammation’, followed by ‘decreases muscle soreness’ (Table 1), which is also supported by the respective free-text answers with the most common physiological reason being ‘decreases swelling/inflammation’. The statement ‘is what I have seen the elite athletes do’ was the lowest rated reason for ALB, AWB, STR and CWT (Table 1). Active, land-based recovery had significantly lower ratings than all other recoveries for the following reasons of use; ‘makes me feel good’, ‘is what I have seen the elite athletes do’, ‘will increase muscle performance’, ‘can improve healing’ and ‘helps me to train/compete hard again in the next session/game’. Cold water immersion rated significantly higher than all other recoveries for the following reasons for use ‘is what I have seen the elite athletes do’, ‘will increase muscle performance’ and ‘reduces swelling and inflammation’. These ratings were somewhat supported by free-text explanations regarding why the athletes believe that each specific recovery method is or is not effective. When the free-text responses for the effectiveness of each recovery method were classified, the highest percentage of responses for ALB and STR were classified as physical benefits, followed by psychological benefits and physiological benefits. In contrast, for water immersion recovery the highest percentage of responses was classified as psychological benefits (Table 2). Table 3 shows the details of the most popular recovery sessions used by athletes after a game/match for each recovery type.

**Table 2:** Number of total responses and the popular response themes from free text answers for the perceived effectiveness of five recovery strategies

	Active, land-based recovery (ALB)	Active, water-based recovery (AWB)	Stretching (STR)	Cold water immersion (CWI)	Contrast water therapy (CWT)
Physical Benefit	*N* = 23 Improves range of movement (8) Loosens (4) Reduces injury (4)	*N* = 41 Improves range of movement (13) Less stress/strain on body (8) Non weight bearing (6)	*N* = 80 Improves range of movement (25) Reduces tightness (23) Loosens (20)	*N* = 5 Reduces tightness (2)	*N* = 2 Reduces stiffness (2)
Physiological benefit	*N* = 18 Warm/cool down (9) Removes lactic acid (3) Blood flow (3)	*N* = 29 Cools (11) Blood flow (7) Pressure (4)	*N* = 23 Blood flow (7) Removes lactic acid (3) Heals muscles (3)	*N* = 35 Reduces swelling/inflammation (16) Cools (11) Removes lactic acid (4)	*N* = 14 Cools (6) Blood flow (3) Reduces swelling/inflammation (3)
Psychological benefit	*N* = 19 Decreases soreness (8) Relax (7) Unwind (4)	*N* = 44 Relax (23) Decreases soreness (10) Freshens (9)	*N* = 51 Decreases soreness (23) Relax (21) Feel better (4)	*N* = 41 Relax (15) Decreases soreness (11) Feels good (10)	*N* = 23 Decreases soreness (9) Relax (6) Feel better (4)
General/unspecified benefit	*N* = 10 It works (3)	*N* = 16 Helps recovery (3) Relatively effective/helpful (2)	*N* = 22 Helps recovery (10)	*N* = 21 Helps recovery (13) Speeds recovery (4) Limited facilities (2)	*N* = 13 Helps recovery (7) Speeds recovery (3)
Sceptical/unsure/neutral	*N* = 13 Don’t feel better (5) Don’t know (3) Sceptical if it works (2)	*N* = 3 Other recoveries better (1) Don’t feel better (1) Don’t know (1)	*N* = 5 Don’t feel better (2)	*N* = 4 Don’t know (2) Don’t feel better (1) Am not convinced of the science of it (1)	*N* = 1 Don’t feel better (1)
Did not answer	*N* = 71	*N* = 29	*N* =38	*N* =31	*N* = 22

**Table 3:** Most popular post-game/match recovery session details (as assessed by statistical mode)

Recovery (number of respondents)	Recovery activity (number of respondents)	Duration (number of respondents)	Timeframe following game/match (number of respondents)
Active, land-based (96)	Walk (69)	10 min (36)	Within 1 h (32)
Active, water-based (89)	Swim (49)	10 min (28)	Within 1 h (29)
Stretching (124)	Static (98)	10 min (56)	Within 1 h (48)
Cold water immersion (71)	Cold water bath immersion (44) to the neck (20)	10 min (53)	Within 1 h (36)
Contrast water therapy (45)	Cold water bath immersion (13) to the shoulders/full body (7); hot shower (32) full body immersion (16)	3 cycles (16) of 1 min cold (16): 1 min hot (20)	Within 1 h (18)

## Discussion

This investigation has identified that a range of recovery strategies are used by athletes across varying team sports and competition levels; and that athletes have varying perceptions of the reason for effectiveness. Fifty-seven percent (mean) of the team sport athletes surveyed performed a recovery after competition and/or training, regardless of competition level, this indicates that most athletes acknowledge that recovery is an integral part of performance and training [16] and supports the hypothesis that most athletes perform a recovery. Massage was used as a recovery strategy by all participating international-level team sport athletes, who also rated massage as the second most effective recovery technique behind sleep, 63% of the national-level athletes also used massage. It is likely that the higher popularity of use of massage by international-level athletes (100% use) and national-level athletes (63% use) in comparison to lower levels of competition athletes (32% state, 45% regional and 22% local) is related to their access to massage therapists who are often members of the support staff.

While stretching was the second most frequently used recovery strategy by the international-level athletes in the current study, it was the most frequently used recovery strategy by all other competition level athletes (national, state, regional and local), partially supporting the hypothesis that stretching would be the most used recovery strategy by all level athletes. Stretching was also rated either the most effective (state, regional and local) or second most effective (national) recovery strategy. Furthermore the athletes that used STR rated it to be significantly more effective as a recovery than the users of ALB, AWB and CWT. The frequent use of stretching by athletes across all competition levels may be attributed to a combination of factors including it can be self-administered, ease of use and accessibility, mainstream popularity, can be performed as a team and its common practice across the fitness and sporting industries. More specifically, stretching requires no equipment, can be performed with minimal space and also has been recommended as a post-exercise recovery across mainstream literature and research for decades [17]. Food/fluid and sleep/nap were also highly used recovery strategies by all levels of athlete (average 79% and 63% use respectively), this is most likely due to these recoveries undertaken as lifestyle choices not as deliberate choices for recovery. This is in contrast to CWI, CWT and AWB strategies that require a deliberate choice to undertake and specialised equipment and facilities, as identified in the free-text responses by the athletes in the current study; with one athlete describing AWB as ‘not practical’, two athletes stating that CWI was ‘not always possible’, and another stating that it was ‘a costly and messy’ recovery strategy.

The main reasons provided by the athletes for the effectiveness of STR were physical or psychological in nature, with the most common response themes being ‘improved range of movement’, ‘decreases tightness’ and ‘decreases soreness’ (Table 3). Research evidence somewhat supports these notions. Stretching has been found to improve range of motion [18, 19] and accordingly decrease tightness of the muscles, although stretching does not appear to be effective for reducing/preventing delayed onset of muscle soreness [20–22]. Thus showing that athletes may not always understand the influence that stretching has upon physical recovery.

The second most effective recovery strategy according to the surveyed athletes of this study was CWI (effectiveness rating 4.3/5). The most commonly provided reason for the effectiveness of CWI was to reduce swelling and inflammation (16 free-text responses and importance rating of 4.2/5; Tables 1 and 2), numerous studies have shown that CWI does not affect inflammation [23, 24]. The athletes also reported ‘relaxes’, ‘cools’ and ‘decreases muscle soreness’ (Table 2) as common reasons for CWI effectiveness for recovery, with importance also placed on improving healing (Table 2). Cold water immersion has been found to cool the body [25] and may also provide an enhanced perception of relaxation [26]. A reduction in muscle soreness is supported by the literature [11, 27] with the mechanism linked to a reduction in neuron transmission speed within the body which decreases experienced pain [28]. This may explain the common analgesic effects reported for CWI [29], and might also improve some sensations associated with tiredness. Notably, CWI received the highest importance rating (3.4/5) of the recovery strategies for the reason ‘is what I have seen the elite athletes do’ (Table 1). The revelation that athletes place importance on whether or not elite athletes are using the CWI recovery also supports the potential belief effect of CWI. While participants indicated that ‘improving healing’ was an important reason associated with the effectiveness of CWI, there is no scientific evidence to support this.

Contrast water therapy was considered to be the third most effective recovery strategy with a score of 4.1/5. The most frequently reported and highest rated reason for CWT use and effectiveness was ‘decreases soreness’. This reason is supported by a review of 13 pooled studies whereby CWT decreased soreness at five time points (>6, 24, 48, 72 and 96 h) in comparison to passive recovery [30]. Athletes also reported that CWT ‘relaxes’ and ‘cools’, with the assumption that the cold water component is responsible for the cooling sensation as noted previously for CWI; and the hot water component is responsible for the sensation of relaxation [31].

All recovery strategies were found to be most commonly used within 1 h of completion of exercise. In contrast CWT has been found to be most commonly used immediately post exercise by elite New Zealand athletes [10] and 12 min post exercise by elite South African rugby union players [13]. The within 1 h post-exercise time frame is mostly likely commonly used as some athletes state they do their recovery at home, so this time frame would be accommodating. It is likely that athletes also believe completing recovery after this time may not be as effective, although Dawson and colleagues [32] found that this may not be the case, finding a ‘next morning’ recovery to be just as effective as an immediate recovery. Athletes mainly undertook recovery strategies of 10 min duration, (CWT approximately 6 min). This study found the most utilised durations for CWT to be 1 min in each temperature for 3 cycles whereas Hing and colleagues [10] found the most used times to be 30 s in cold, 1 min in hot, for 3 cycles. This study found the most utilised duration for CWI to be 10 min, although Van Wyk and Lambert [13] found CWI of 2 x 3 min immersions to be most commonly used. Versey and colleagues [33] state that CWI needs to be 5-15 min and CWT up to 15 min for optimal results. This study has found differences between levels of immersion used for CWI and the cold component of CWT. The differences though are minimal with the regions being neck, shoulder and whole body all being very similar when considering water immersion. Halson [34] states that whole body immersion be used to increase effectiveness of water immersion protocols.

While this study identifies the use of recovery strategies by team sport athletes and their perceptions of recovery strategies and effectiveness, this study has some limitations. Similar to Venter [12] the assumption that participants’ responses were accurate and the potential influence of other athletes when completing the survey may have influenced the results. Misinterpreting information when completing the survey may also have occurred. While five competition levels were represented in the sampling, most athletes were based in northern Queensland and therefore results are indicative of that region, and may not represent the whole of the country. Another limitation is the difference between the physiological demands of the sports that the participants played, although all of the sports represented by regional, state, national and international athletes were also represented by local athletes. Future research should continue to look at the usage of popular recovery methods, including the use of mental techniques [35] within Australia (coverage of multiple states and cities) and their reasons for use.

## Conclusion

In summary, to our knowledge this is the first study to explore the post-exercise recovery practices of Australian team sport athletes and which identifies and explains their perceptions and preferential use of particular strategies. It was found that international athletes utilise massage the most and consider sleep and massage to be the most effective recovery strategies. This may be due to greater access to massage practitioners at the elite level of team sports as all other levels of athlete utilised stretching the most and considered it the most or second most effective recovery strategy. When asked to rate the five discussed recoveries, STR was rated the most effective with ALB considered the least effective by its users. Laziness and time constraints were the main reasons provided by the 43% of athletes who did not undertake recovery. This study determined that athletes are aware of how they feel following the use of recovery and they use recovery based on their perceptions, but may not be able to identify why a recovery method is effective/ineffective. This study also highlights how the perceptions of athletes do not always align with scientific evidence. It is suggested that the availability of particular recovery strategies may also impact upon recovery strategy selection. It is encouraged that athletes and coaching staff are informed about the effects different recovery strategies have upon the body to ensure recovery strategies are selected and implemented for the correct reasons.

## Abbreviations

ALB: Active, land-based recovery
AWB: Active, water-based recovery
CWI: Cold water immersion
CWT: Contrast water therapy
STR: Stretching
